# Development of a core outcome set and outcome measurement set for physiotherapy trials in adults with Bronchiectasis (COS-PHyBE study): A protocol

**DOI:** 10.1371/journal.pone.0263695

**Published:** 2022-02-08

**Authors:** Hayat Hamzeh, Sally Spencer, Carol Kelly

**Affiliations:** 1 Faculty of Health, Social Care & Medicine, Edge Hill University, Ormskirk, United Kingdom; 2 Respiratory Research Centre, Edge Hill University, Ormskirk, Lancashire, United Kingdom; 3 Health Research Institute, Edge Hill University, Ormskirk, Lancashire, United Kingdom; Kwangwoon University, REPUBLIC OF KOREA

## Abstract

**Background:**

Bronchiectasis is a chronic respiratory disease characterised by airways widening and recurrent infections, resulting in episodes of chronic cough, sputum expectoration, and dyspnoea. This leads to deterioration in daily function, repeated hospital admissions and poor quality of life. The prevalence and mortality related to bronchiectasis is increasing worldwide with growing economic burden on healthcare systems. Physiotherapy for bronchiectasis aims to decrease accumulation of sputum, dyspnoea, and improve exercise capacity and daily function. A robust evidence base to support physiotherapy in bronchiectasis is currently lacking. This is partly because of inconsistency and poor reporting of outcomes in available studies.

A core outcome set is the minimum acceptable group of outcomes that should be used in clinical trials for a specific condition. This decreases research waste by improving consistency and reporting of key outcomes and facilitates the synthesis of study outcomes in systematic reviews and guidelines.

The aim of the study is therefore to develop a core outcome set and outcome measurement set for physiotherapy research in adults with bronchiectasis. This will ensure outcomes important to key stakeholders are consistently used and reported in future research.

**Methods and analysis:**

This project will use the COMET Initiative and COSMIN guidelines of core outcome set development and will include three phases. In the first phase, a comprehensive list of outcomes will be developed using systematic review of reported outcomes and qualitative interviews with patients and physiotherapists. Then consensus on key outcomes will be established in phase two using a Delphi survey and a consensus meeting. Finally, in phase three, we will identify appropriate instruments to measure the core outcomes by evaluating the psychometric properties of available instruments and a stakeholders’ meeting to establish consensus.

**Ethics:**

The study was reviewed and has received ethical approval from the health-related Research Ethics Committee- Edge Hill University (ETH2021-0217).

**Registration:**

This study is registered with the COMET database. https://www.comet-initiative.org/Studies/Details/1931.

The full systematic review protocol is registered in PROSPERO under the number CRD42021266247.

## Background

Bronchiectasis is a chronic respiratory disease characterised by widening and thickening of the airways, leading to accumulation of secretions and recurrent infections [1,2]. Bronchiectasis has a negative impact on quality of life, including difficulty completing daily activities, social embarrassment, anxiety, and sleep deprivation [3,4]. The prevalence of bronchiectasis has increased globally in recent years [5], causing a substantial economic burden of approximately $36,000 annually per person as calculated in US and Spanish cohorts [6]. Cost is attributed to hospitalizations, outpatient service use, physiotherapy and rehabilitation, and long-term medications [7,8]. UK bronchiectasis-related mortality is more than twice that of the general population [9], while 5 years mortality is 12.4% in European population [10].

Physiotherapy for bronchiectasis encompasses a group of interventions aimed at improving symptoms and functionality, including airway clearance, pulmonary rehabilitation, exercise and breathing training [11]. These are usually delivered via customised interventions involving teaching self-administered airway clearance techniques and a home exercise program [12].

While physiotherapy is recognised as a core element of bronchiectasis care [12–14], it currently lacks high quality evidence of its effectiveness [15,16]. This is partly attributable to difficulties in aggregating data from clinical trials in systematic reviews, due to inconsistent outcome reporting and variation of measurement instruments, with some important outcomes, such as exacerbation, hospitalisation and side effects, commonly missing [17–22]. This is particularly relevant for bronchiectasis research where there are very few large-scale trials and therefore the synthesis of results from smaller trials is important for assessing evidence of effectiveness. Consequently, using COMET (Core Outcome Measurement in Effectiveness Trials) methodology to standardise outcome reporting is important for improving the design of future trials [23]. Spargo and colleagues (2019) [24] has developed a core outcome set for bronchiectasis, it included all types of intervention and the expert group of the Delphi study included mainly physicians, which limits its validity for use in physiotherapy specific trials. The COS included 18 outcomes, which is a large number to be used in trials and systematic reviews. Defining OMIs for bronchiectasis studies were not included in the study, which limits the usability of its results by researchers and clinicians.

### Aims and objectives

A core outcome set (COS) comprises the minimum agreed outcomes that should be measured and reported in trials for a given health condition [25]. Thus, establishing not only ‘what’ should be measured in this area of research, but also ‘how’ to measure it. The main aim of this study is therefore to work with patients and healthcare professionals to develop a COS for use in trials examining the benefits of physiotherapy for adults with bronchiectasis. This core outcome set focuses on adults as children may have different problems and needs that require a separate set of outcomes. The main objectives are:

1. To identify a list of outcomes currently reported in physiotherapy trials for bronchiectasis
2. To evaluate consistency in outcome reporting in published trials and trial protocols.
3. To explore the important outcomes for stakeholders, including patients and physiotherapists
4. To establish consensus among researchers and stakeholders on the most important outcomes to be included in the COS.
5. To develop a Core Measurement Set (CMS) by identifying outcome measurement instruments (OMIs) for reporting the COS.

## Methods

**Fig 1:**
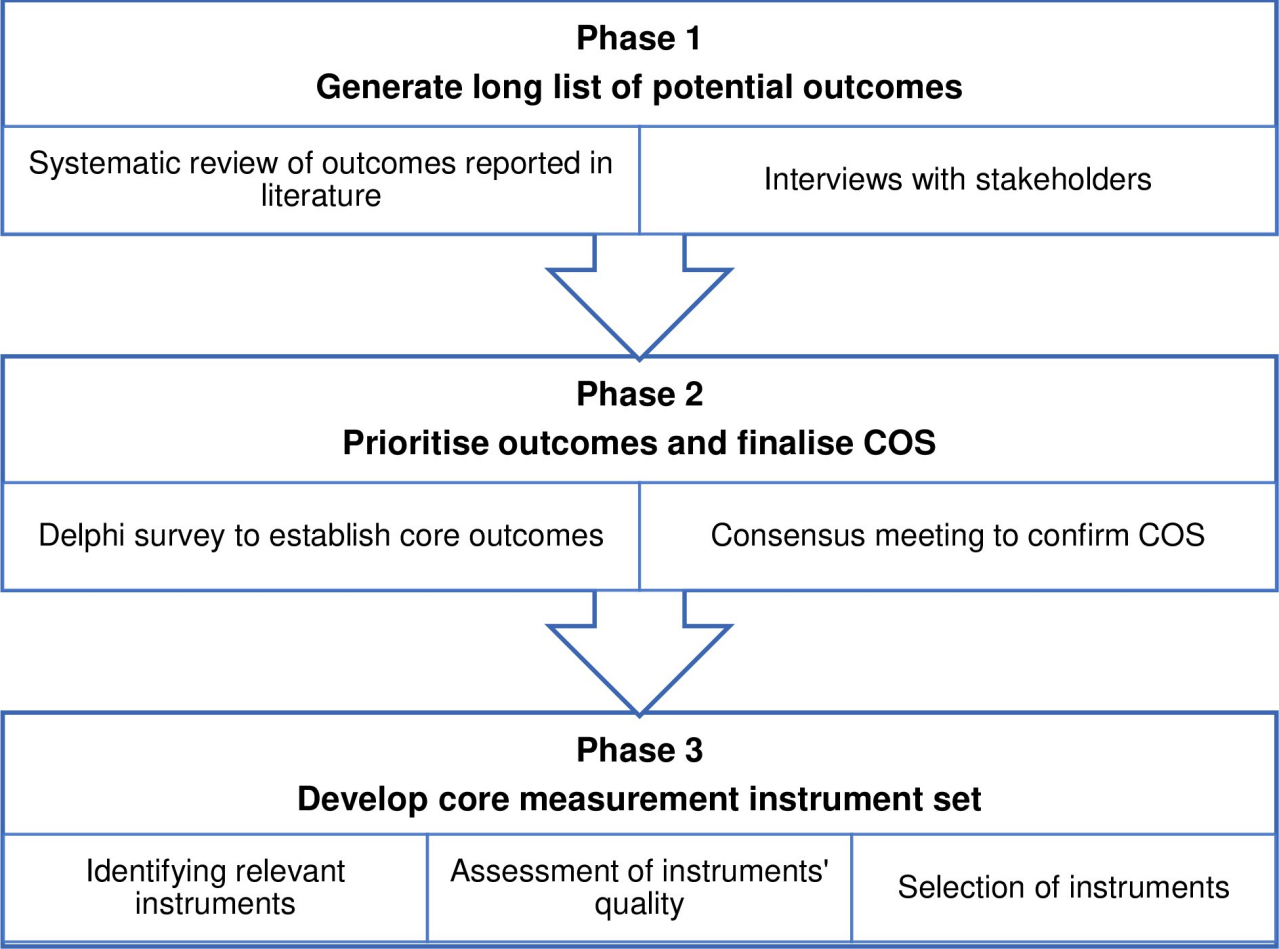
COS development structure.

The study design is based on recommendations developed by COMET and COSMIN (Consensus-based Standards for the selection of health Measurement Instruments) standard setting initiatives. COMET has supported the development of hundreds of COS’s with over 800 currently registered on their online database [26]. Protocol methods are based on the Core Outcome Set—Standardised Protocol Items (COS-STAP) [27] and the 11 minimum standards for COS development (COS-STAD) [28] shown in Table 1. The three-phase study design is shown in Fig 1 and the protocol is registered on the COMET database.

**Table 1:** Developing COS process based on the COMET COS-STAD [28].

Domain	Standard number	Methodology	Application in the proposed study
**Scope specification**	1.	The research or practice setting for the COS	Physiotherapy effectiveness trials
	2.	The health condition covered by the COS	Bronchiectasis
	3.	The population covered by the COS	Adults (18+)
	4.	The interventions covered by the COS	All physiotherapy interventions, including airway clearance, positive expiratory pressure devices, and pulmonary rehabilitation
**Stakeholders involved**	5.	Those who will use the COS in research	Researchers interested in bronchiectasis
	6.	Healthcare professionals with experience of patients with the condition	Respiratory physiotherapists
	7.	Patients with the condition or their representatives	Adult patients with bronchiectasis
**Consensus process**	8.	Long-list of outcomes considered by stakeholders.	Systematic review and interviews with patients and clinicians.
	9.	Scoring process and consensus definition.	Delphi scoring using a nine-point Likert scale (1–3, not important; 4–6, important; 7–9, critically important) Consensus criteria: score of 7+ per item from ≥70% of respondents
	10.	Criteria for including/eliminating outcomes.	inclusion: outcomes scored ‘critically important’ from ≥70% AND ‘not important’ from < 15% of participants exclusion: outcomes scored ‘critically important’ from less than 50% of participants
	11.	Avoiding language ambiguity in the description of outcomes.	Plain language version will be available, informed by interviews and pilot-tested with patients.

### Stakeholder involvement

The COS-PHyBE study aims to produces a COS that is meaningful and inclusive for all stakeholders. Therefore, patient representatives and respiratory physiotherapists were invited to be research advisors, who will be involved in designing, piloting, and recruitment for the study. The research team also includes researchers and systematic reviewers with interest in bronchiectasis research.

### Phase one: Generating long-list of potential outcomes

We will create a long-list of potential outcomes using a systematic review of academic literature and semi-structured interviews of patients and physiotherapists.

#### Systematic review of academic literature

We did not identify similar or ongoing systematic reviews on the international prospective register of systematic reviews (PROSPERO). We will undertake a systematic review of randomised control trials (RCTs) and protocols for RCTs investigating the benefits of physiotherapy interventions for bronchiectasis. We will limit the search to RCTs as the purpose of the COS-PHyBE study is to influence the design of future RCTs. The protocol for this systematic review is registered on PROSPERO (CRD42021266247).

We will search PubMed, EMBASE, CINAHL, Pedro, and the Cochrane Central Register of Controlled Trials (CENTRAL) from their inception to current date. We will also search protocol registries, reference lists, dissertations, and relevant conference proceedings for relevant trials. The search will be limited to trials published in the English language due to limited translation resources for publications in other languages.

Randomised and quasi randomised clinical trials of any physiotherapy intervention for bronchiectasis will be included. Trials will be excluded if the study population included children (<18 years) or mixed respiratory populations (e.g., participants from multiple respiratory disease cohorts).

Two reviewers will independently perform study selection and data extraction, with disagreements resolved by consensus. We will screen titles and abstracts for eligibility and confirm using the full text. Study selection will be reported using the Preferred Reporting Items for Systematic Reviews and Meta-Analyses (PRISMA) guideline. We will extract study population characteristics, all reported outcomes, their definitions and domains, the OMIs used to measure them, and the measurement timepoints.

The COS taxonomy will be used to classify outcomes. The 38 outcome domains cover the core areas of death, physiological outcomes, life impact, resource use, and adverse effects [29]. Results will include a narrative description of the outcomes and their categories, variation in outcome definitions, different interventions, and differences in outcomes across different interventions.

#### Semi-structured stakeholder interviews

The aim of this phase is to identify outcomes important to patients who receive the treatment and physiotherapists who assess these outcomes in daily practice. This will ensure that the long-list will include, in addition to outcomes identified in the literature, the most important outcomes to stakeholders, to enhance utility and uptake of the final COS [23].

We will interview 6–12 patients and the same number of physiotherapists, considered sufficient to achieve data saturation, whereby no new outcomes are identified [30]. sample will include adult patients diagnosed with bronchiectasis and previously treated by physiotherapy, as well as physiotherapists clinically involved in bronchiectasis care for at least one year. To ensure a broad range of views are represented we will recruit participants through online channels. This will assist recruiting participants from multiple countries and settings, and with different backgrounds and experiences.

Patients will be recruited through the European Lung Foundation (ELF) and via patient support groups on social media. Physiotherapists will be recruited through the Association of Chartered Physiotherapists in Respiratory Care (ACPRC), and other professional networks. Participants unable to complete an interview in English will be excluded.

Interviews will be done remotely via video conferencing or telephone. Participant information and informed consent forms will be sent to participants before the interview date and consent will be recorded during the interview. A semi structured interview format will be followed, and the main theme of the interviews will be exploring outcomes important to participants. Interview guides will be prepared considering similar qualitative studies and results of the systematic review. Interview guides were prepared for each group and validated by patients and physiotherapist representatives (S1 Appendix).

The interviews will be digitally recorded then transcribed verbatim. Transcripts will be analysed using thematic analysis methods [31], supported by NVivo qualitative data analysis software version 12 [32]. The analysis will aim to identify and explain important outcomes and describe them using participants’ language. Outcomes which are mentioned directly or indirectly will be extracted from the qualitative data. Thematic analysis will be guided by the COMET outcomes classification as an analytical framework. Frequency that each outcome is mentioned and justifications for its value to participants will be used to determine the importance of outcomes. A list of all outcomes identified from interviews will be prepared by the end of analysis. The Consolidated criteria for Reporting Qualitative research (COREQ) will be used to report the qualitative study [33].

### Phase two: Prioritise outcomes and finalise COS

Consensus on core outcomes will be achieved using a Delphi survey, followed by a stakeholder meeting to establish consensus.

#### Delphi survey

COS studies commonly use Delphi surveys to achieve consensus on the most important outcomes to include [23,34]. The technique ensures all participants make an equal contribution and involves multiple anonymised prioritisation rounds [35]. A modified Delphi design is recommended in COS development, where the list of outcomes in phase 1 comprises the first round [23]. This modified Delphi study will involve two rounds conducted using the onlinesurveys.ac.uk interface (JISC, Bristol, UK) to maximise participation and enhance credibility [34]. Each round will take approximately 3 weeks and participants will be promoted with weekly reminders.

*Formulation of the survey items*. The two lists of outcomes produced from the systematic review and the interviews will be merged to produce the long outcomes list. This list will then be discussed with the research advisors to merge similar outcomes and decide the list of outcomes to be taken into the Delphi survey. Using the COMET taxonomy the list will be grouped into outcome domains [23]. A definition for each outcome in simple language will be prepared and verified by patient representatives. The final survey will then be pilot tested by research advisors to ensure clarity and feasibility before data collection.

*Sample and recruitment*. Adults aged 18+ with bronchiectasis, physiotherapists, and researchers able to complete a survey in the English language will be asked to participate. Consent to participate will be implied by completion of the survey.

There is no required sample size for a Delphi study, but good representation of each group is essential [23,34]. Therefore, a pragmatic approach will be taken, and all interested participants will be included in the study. Having different group sizes may downgrade the voice of smallest group. Hence, data analysis will be performed separately for the three groups, this will allow for intra and inter group variability to be explored and for equal group’s representation in the process.

Recruitment will be facilitated through multiple channels to focus on maximising international participation and representation of various demographic populations. We will recruit through patient networks (such as the European Lung Foundation), professional organisations (such as The International Confederation of Cardiorespiratory Physical Therapists (ICCrPT)), research networks (such as the European Multicentre Bronchiectasis Audit and Research Collaboration (EMBARC)), other professional organisations and societies, patients’ groups on social media, and contacting key researchers identified in phase 1. A Lay language summary explaining the study objectives will be prepared using documents from the COMET Patient Participation, Involvement and Engagement group.

*First round*. A nine-points Likert scale will be used to score items according to their importance and an option of ‘unable to score’ will be added for each item. 1 to 3 signifies an outcome is of limited importance, 4 to 6 important but not critical, and 7 to 9 critical. This scoring system is widely used in COS studies and is recommended by COMET and GRADE [25]. Participants will be asked to rate each outcome’s importance according to their own experiences and opinions. One open ended question will be added at the end of the first-round questionnaire to capture any new suggested outcomes.

*Second round*. All outcomes will be carried through to second round in addition to new suggested outcomes from round one. New suggested outcomes will go through a review process; they will be carried out to next stage if they are genuinely new and can’t be included under any other outcomes. For each outcome, collective scores obtained from each group in round one will be displayed alongside the participant’s personal previous score, scores will be accompanied by graphs to enhance visual presentation. Participants will be asked to consider previous scores and rate each item using the same Likert scale. All participants in round two will be asked if they are interested in attending the subsequent consensus meeting.

Consensus levels will be defined as following:

1. Included: a score of 7 to 9 from more than 70% of participants and a score of 1 to 3 from less than 15% of participants in all groups. These items will be included in the final COS.
2. Excluded: a score of 7 to 9 from less than 50% of participants in all groups. These items will be discarded from the final COS.
3. No consensus: items which does not achieve the inclusion or exclusion criteria. These will be taken into the consensus meeting for discussion and final voting.

*Missing data*. The rate of missing and incomplete responses will be reported with the results of the Delphi study. If a participant did not complete all rounds, available responses will be included in analysis. As analysis will be performed separately for each item, incomplete responses will not be discarded, and available items will be included in the analysis. To test if missing data affect representativeness, the first-round participant profiles will be compared to participants who completed both rounds.

#### Consensus meeting

Following the Delphi survey, a consensus meeting will be held to discuss the results and recommend the final COS outcome domains. A pragmatic representative sample will be invited from participants who expressed interest in the second Delphi round. Outcomes which achieved no consensus in the Delphi survey will be subjected to voting. A modified nominal group technique will be used, which is a structured group discussion that involves generating, defining, and ranking items to reach consensus while limiting individual dominance [36]. This method will involve a series of discussing outcomes, nominating most and least important outcomes by each participant, anonymous voting on outcomes, discussion of voting results and agreeing final COS. Nine point Likert scale will be used for voting, and the same consensus criteria of the Delphi survey will be applied. Voting results will be presented by the end of meeting for final COS agreement.

### Phase three: Development of a core measurement instrument set

The COSMIN/COMET guidelines for establishing a set of measurement instruments for the COS [37] will be followed (Table 2). The process includes a systematic review followed by a stakeholder’s meeting.

**Table 2:** Proposed CMS development process based on COSMIN/COMET recommendations [37].

Recommendation	Proposed action
**Step 1. Conceptual considerations**	• Target population: adults with bronchiectasis • Intervention: physiotherapy • Outcomes: All outcomes included in the COS developed in phase 2
**Step 2. Identify existing outcome measurement instruments**	Outcome measurement instruments used in the literature will be identified as part of the systematic review in phase 1.
**Step 3. Quality assessment of outcome measurement instruments**	• Search for available reviews of psychometric properties of each instrument. • Systematic review will be undertaken for instruments where no previous reviews are available
**Step 4. Selection of outcome measurement instruments for the COS**	• Stakeholder’s meeting to approve instruments selection based on the systematic review results.

#### Step 1. Conceptual considerations

The outcomes for which CMS will be identified in phase two of the study. Scope of the CMS is detailed in Table *2*.

#### Step 2. Identify existing outcome measurement instruments

Existing instruments will be identified as part of the systematic review of available trials in phase one. Information regarding instruments used in trials will be extracted, they will appropriately be tabulated and classified according to relevant outcomes. Another search will be run to identify any available systematic reviews which evaluated psychometric properties of these instruments. The COSMIN Database of systematic reviews of outcome measurement instruments and PubMed Central will be searched for this purpose.

*Systematic review of outcome measurement instruments*. If no systematic reviews can be identified for a certain instrument, or if the available reviews are outdated or of poor quality, a separate systematic review of outcome measurement properties will be conducted. The review will be performed according to the COSMIN/COMET guidelines [35]. We will search PubMed, EMBASE, and CINAHL from inception to current date. The search will be limited to trials published in the English language. Studies which investigated psychometric properties of the instruments in bronchiectasis will be included.

Two reviewers will independently perform study selection and data extraction, with disagreements resolved by consensus. We will extract study population, instrument definition, results of the measurement properties assessed, evidence on interpretability and feasibility (e.g., scores description, use of devices, floor and ceiling effects, minimal important change or difference, ease of standardization and calculation, completion time). For PROMs, mode of administration, original language and available translations will additionally be extracted.

#### Step 3. Quality assessment of outcome measurement instruments

Evaluation of the quality of the measurement properties for each OMI will be assessed using the COS-MIN tool which was developed by COSMIN and COMET [37]. This tool is recommended to evaluate the psychometric properties of patient reported, clinician-reported, and performance-based outcome measurement instrument, in addition to laboratory values. Rated measurement properties include different forms of validity (content, structural, criterion, cross cultural), internal consistency, reliability, measurement error, hypotheses testing, and responsiveness. Each measurement property will be rated as positive, intermediate, or negative. Then overall quality of OMI will be rated from high to unknown according to the recommended criteria.

#### Step 4. Selection of outcome measurement instruments for the COS

A table of the psychometric properties and quality for each OMI will be prepared. For each outcome, OMIs will be recommended based on their quality assessment. An OMI will be recommended only if it meets the following minimum COS inclusion requirements: at least high-quality evidence of good content validity, high quality evidence of internal consistency, test-retest or inter-rater reliability (if applicable), and if it seems feasible.

Where there is missing data regarding psychometric properties for an OMI, we will recommend further validation work. These will be added to the final list as provisional outcome measurement instruments.

*Stakeholder’s meeting*. The aim of the final stakeholders meeting will be to ensure transparency of the process and approve the final CMS. We will invite key researchers, physiotherapists, and patient representatives to attend. Participants will discuss results from the systematic review and recommendations for the final list of instruments and appropriate time points for measurement. A single OMI will be chosen for each outcome, this will be based on discussion of feasibility aspects in case two instruments has similar quality criteria.

### The status and timeline of the study

The first phase of the study; systematic review of outcome reporting is currently ongoing. The expected end date of the study is January 2024

### Limitations of the study design

Although all efforts will be made to encourage international participation in the study, this will be limited by the inability to conduct the study in languages other than English. Using stakeholders meeting only to determine the core measurement instruments may risk bias towards opinions of individuals present at the meeting. We anticipate that our dissemination strategy will lead to satisfactory uptake of the COS with relevant researchers, but it is not guaranteed that it will be implemented in future research.

### Dissemination plan

The study will be published in peer reviewed journals. The results from this research will be presented at meetings, seminars and symposiums and at relevant conferences. They will also be shared with all researchers who participated in the project and experts in the field. The results will also be presented to interest groups and organisations, such as EMBARC, ELF, ERS, ICCRPT, ACPRC, BTS. We will additionally publicise the COS updates and results via the project’s Twitter page. Lay language summaries will be shared with relevant societies and patient groups for dissemination through their websites and social media.

## Supporting information

S1 Appendix Interview guides for the semi-structured stakeholder interviews. (DOCX)
