# Supplementary material for: Development of a core outcome set and outcome measurement set for physiotherapy trials in adults with Bronchiectasis (COS-PHyBE study): A protocol
Source: PLoS One. 2022 Feb 8;17(2):e0263695. doi: 10.1371/journal.pone.0263695 (PMC8824374; doi:10.1371/journal.pone.0263695)
Supplement: S1 Appendix — (DOCX) [file pone.0263695.s001.docx]

### Topic guide qualitative interviews – patients

***Introduction (5 mins)***

- Interviewer name and background
- Explain expected aims, sensitive subjects and duration of interview and confidentiality.
- Confirm consent to qualitative interview and recording.

***Background (5 min)***

- Please confirm your age.
- How long have you been living with bronchiectasis?
- what treatments you’ve been taken? What form of physiotherapy have you tried?

***Interview questions (40 mins)***

- Describe your experience with physiotherapy for your condition.
- What do you hope will change in your health and life after receiving physiotherapy?
- OR
- Which of your challenges do you think physiotherapy may help with?
- Think about one of your physiotherapy sessions. How did you know it was successful?
- What do you think should be measured in research as a marker of physiotherapy benefit?

Probe: Can you define that to me please? Or give an example of it.

- When do you think these should be measured to reflect change in your condition?
- How do you think these should be measured?
- OR
- You've mentioned … how do you think we can measure this?
- Would you be able to prioritise the importance of these for you now?

Optional

- Would these outcomes be different if you have a different method of physiotherapy?

***Closing remarks (5 mins)***

- Is there anything do you want to add that might be important for me to hear?
- If you remember anything in the future, or if you have any questions regarding this research. Please email me.
- Thank the participant.

### Topic guide qualitative interviews – physiotherapists

***Introduction (5 mins)***

- Interviewer name and background
- Explain expected aims, sensitive subjects and duration of interview and confidentiality.
- Confirm consent to qualitative interview and recording.

***Background (5 min)***

- Tell me about your background and experience in respiratory physiotherapy
- What about your experience with bronchiectasis? What treatments do you use for this population?

***Interview questions (40 mins)***

- What are the outcomes/goals do you usually use for bronchiectasis cases? Why?
- Would you be able to prioritise the importance of these for you now?
- What are the outcome measures do you prefer to use with these cases? Why?
- What timelines do you think these outcomes should be measured at?
- Would you use a different set of outcomes with different treatment techniques?
- What outcomes do you think we should use in research in this area?
- Do you think we should use specific outcomes for bronchiectasis that are different from other lung diseases?

***Closing remarks (5 mins)***

- Is there anything do you want to add?
- If you remember anything in the future, or if you have any questions regarding this research. Please email me.
- Thank the participant
